# The Segmentation and 3D Reconstruction of Teeth, Root Canals, and Alveolar Bones in CBCT Images Based on AVIZO Software

**DOI:** 10.1155/ijod/7377174

**Published:** 2026-02-19

**Authors:** Yan Liu, Chunshen Li, Pengyu Chen, Yunshan Zhao, Liang-qiu-yue Zhong, Zhongyi Xiao, Xi Chen

**Affiliations:** ^1^ Department of Stomatology, The First Affiliated Hospital of Xi’an Jiaotong University, 277 Yanta West Road, Xi’an, 710061, Shaanxi, China, xjtu.edu.cn

**Keywords:** 3D reconstruction, alveolar bone, root canal, segmentation, tooth

## Abstract

**Background:**

This study utilized AVIZO software and manual segmentation techniques to extract individual teeth, root canals, and alveolar bone structures from cone beam computed tomography (CBCT) images. A three‐dimensional model suitable for personalized treatment planning was constructed, which holds significant implications for clinical diagnosis and treatment.

**Methods:**

We collected 576 CBCT dental images. Based on AVIZO software, the tooth, root canal, and alveolar bone were segmented and 3D reconstructed by image calibration, image noise reduction and edge enhancement, manual labeling, and other methods.

**Results:**

Our approach enables segmentation and reconstruction, allowing for quantitative analysis of 3D reconstructed teeth and root canals, including tooth 3D volume size, dip angle, azimuth angle, and shape factors. Three‐dimensional volume range: 0.31–1.11 cm^3^, tooth dip angle range: 2.02–49.39, tooth azimuth range: −158.38−176.36, tooth shape factor range: 3.74–9.59, and root canal shape factor range: 4.93−17.64.

**Conclusions:**

We successfully achieved segmentation and three‐dimensional reconstruction of teeth, root canals, and alveolar bone. Based on the reconstruction results, we obtained corresponding quantitative data, providing objective and quantitative evidence for developing personalized treatment plans.

## 1. Introduction

With the development of computer technology and digital oral medicine, cone beam computed tomography (CBCT) has become more and more important in the diagnosis and treatment plan of oral diseases due to its advantages of clear imaging and low radiation dose [1]. Segmentation of an individual tooth of CBCT images is a necessary prerequisite for establishing an effective computer‐aided diagnostic system in clinical applications [2, 3]. The segmentation of each tooth from the CBCT image allows detailed analysis of its shape, position, and relationship with the surrounding tissue structure [4]. The construction of a complete 3D dental model can assist orthodontic and implant surgery making treatment plans, which is crucial for digital oral diagnosis and treatment plans [5–7]. Accurate segmentation of individual tooth from CBCT images can help diagnose caries, pulpitis, and plan for filling [8], root canal treatment, and crown repair [9]. By segmenting the root canal, the complexity can be analyzed to help dentists judge the difficulty of root canal treatment [10]. By segmenting the alveolar bone, accurate anatomical information can be provided for orthognathic surgery, and dentists can simulate and evaluate different treatment modalities in the virtual environment, which can significantly improve the efficiency and accuracy of the surgery [11].

With the development of computer vision technology, various automatic image segmentation methods have been introduced. The existing automatic dental segmentation algorithms can be roughly divided into tooth segmentation algorithm based on threshold value [12], tooth segmentation algorithm based on region growth [13], tooth segmentation algorithm based on level set model [14], and tooth segmentation algorithm based on deep learning method [15]. The threshold‐based segmentation algorithm is a traditional digital image processing algorithm. Its principle is to select a suitable threshold value as the boundary of segmentation and to separate the target and the background region. The region growth algorithm, which is applied to partial image segmentation, is a traditional region‐based image segmentation algorithm. The basic idea of this method is to aggregate similar pixels together to form a region. The principle of the level set method is to express the lower one‐dimensional function as the zero‐level set function of the higher‐dimensional function, and the final evolution result depends on the position of the higher‐dimensional function in its zero‐level set. Although the above methods significantly reduce the time required for tooth segmentation, the above methods present a challenge for accurate segmentation of an individual tooth and a root canal for the following reasons: (1) In CBCT images, the boundary between the alveolar bone and tooth root around teeth is not clear, the grayscale is very close, and the tooth shape and posture are different, which will lead to insufficient and excessive segmentation, and the reconstructed 3D tooth model will lose part of the information or cannot segment some structures [16]; (2) the gray range of the tooth and root canal at the junction is also very similar, and the diversity and complex shape of the root canal will affect the accuracy of its segmentation [17]; (3) in the normal state of biting, the upper and lower teeth will have a common contact boundary, which will prevent accurate tooth segmentation [18]. Therefore, accurate segmentation of individual tooth, root canal, and alveolar bone from CBCT images is a challenging task. In recent years, fully automated learning methods have made significant progress, driven by the introduction of deep learning [19]. Although automatic tooth segmentation is an efficient algorithm to automatically extract 3D tooth and substructure models, this method often requires a lot of manpower and material resources [20], and the network model has many parameters and high model complexity [21, 22]. The network model is built on a large number of training data, and the lack of data is also a key problem affecting the precision of tooth segmentation [23].

AVIZO software has demonstrated exceptional efficacy in the realm of medical image processing, particularly in tooth segmentation tasks [24]. Its primary advantages can be summarized as follows: (1) It supports a variety of multimodal data types, including CT, Micro‐CT, and MRI, enabling the precise representation of the three‐dimensional structure of teeth and surrounding tissues [25]; (2) utilizing volume rendering and surface reconstruction technologies allows for accurate segmentation and high‐resolution reconstruction of dental anatomical details such as crowns, roots, and pulp cavities [26]; (3) the software offers specialized segmentation tools that encompass a wide range of functions [27]. Threshold segmentation: This technique utilizes the gray‐level differences between teeth and surrounding tissues (such as bone and soft tissue) to achieve rapid initial separation. Region growing: This method is particularly effective for distinguishing dentin from enamel. Machine learning module (integrated with plugins or scripts): This component enhances segmentation efficiency in complex cases, such as dental deformities and crowded dentition, through model training. Interactive editing tools: These tools facilitate manual correction of segmentation results, addressing potential limitations inherent in automated algorithms. It should be clarified that all functions of the AVIZO software, including thresholding, region growing, filtering, and image enhancement tools, are used solely as initial auxiliary means. Researchers first employ these tools to obtain an approximate contour, but the final precise contour lines for all tissues are manually traced, refined, and ultimately confirmed layer by layer across three orthogonal views (axial, coronal, and sagittal). Image enhancement tools optimize visibility in low‐contrast regions, allowing operators to manually trace boundaries based on clear visual references.

CBCT imaging process will introduce some noise due to various reasons, and the use of appropriate noise reduction methods can remove and reduce the noise factor. AVIZO software provides Gaussian filter, median filter, bilateral filter, nonmean filter, Sobel filter, symmetric nearest neighbor (SNN), and other filtering methods to optimize image quality, enhance feature extraction capabilities, and significantly improve the accuracy and efficiency of the segmentation algorithm, which has important scientific and clinical application value in dental image segmentation [28]. Based on these capabilities, the software uses different filtering methods to improve image segmentation accuracy and then performs segmentation and reconstruction of tooth morphology, root canal system, and alveolar bone to assist in the development of dental implants, orthodontics, or maxillofacial surgery.

In this study, the segmentation of teeth, root canals, and alveolar bone was performed manually by trained researchers, constituting a core step. AVIZO software served as a robust supporting platform throughout the process rather than a fully automated segmentation system. Specifically, we employed the software’s threshold segmentation, region growing, image filtering, and enhancement tools as initialization and auxiliary methods. However, the final contours of all tissue structures underwent meticulous manual refinement, boundary confirmation, and correction. Thus, AVIZO functioned as an integrated tool combining visualization, image preprocessing, interactive annotation, 3D reconstruction, and quantitative analysis. While this workflow relied to some extent on operator expertise and was time‐consuming, it fully leveraged the anatomical knowledge advantages of clinical practitioners. However, unlike previous segmentation methods, we segmented each tooth, root canal, and alveolar bone structure without relying on a large of data for network model training. We can apply to the tooth segmentation of patient in the clinic, which is crucial for dentists to make detailed treatment plans.

## 2. Materials and Methods

This study was approved by the Ethics Committee of the First Affiliated Hospital of Xi’an Jiaotong University, China (no. XJTU1AF2022LSK‐027).

### 2.1. Data Collection

**Figure 1:**
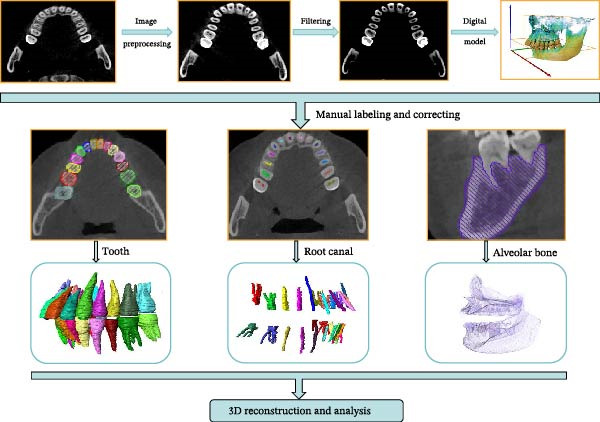
The framework used in this study.

All analytical data originated from one patient who met all of the following criteria: (1) Adults aged 18 to 55 years; (2) no dental restorations (including crowns and fixed bridges), implants, or fillings in the oral cavity. (3) No prior orthodontic treatment or in the initial state prior to orthodontic treatment initiation. (4) Possesses a complete set of 28 permanent teeth, with no deciduous teeth present, and has had the third molars (wisdom teeth) extracted or is missing them. This patient underwent a comprehensive oral CBCT scan. We then systematically reconstructed 576 distinct two‐dimensional images from the raw three‐dimensional dataset of this scan. These 576 CBCT images were primarily used to establish a source database for slice selection, aiming to identify the optimal slice that clearly displays the anatomical structures of dental hard tissues, pulp chambers, and root canal systems. The collected 576 CBCT scans are not publicly available in the Department of Stomatology of the First Affiliated Hospital of Xi’an Jiaotong University. In this study, KaVo CBCT was taken in Germany, and AVIZO (2019) software platform was used for 3D reconstruction and analysis. The overall methodology is succinctly described in Figure 1.

### 2.2. CBCT Imaging Principle

**Figure 2:**
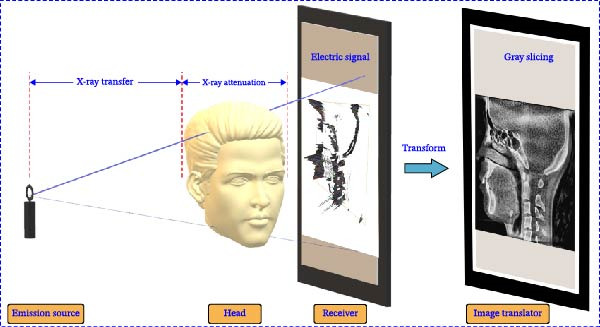
Working principle of CBCT image acquisition.

X‐rays will produce different attenuation effects after passing through different tissues of the human body. The detector converts the attenuated X‐ray signal into an electrical signal and then into a perspective image, as shown in Figure 2 [29]. These images can be processed by software to show sagittal, coronal, and axial plane tooth images. CBCT uses a 5 mA current and 120 KV voltage to perform a single 360° rotational scan around the patient’s head with a thickness of 0.2 mm per scan, which greatly improves image accuracy and captures anatomical details of oral structures.

### 2.3. AVIZO Software Description

AVIZO software can be used to explore science data obtained from CT scans, MRI, and other technologies. The software provides tools for visualization, measurement, 3D reconstruction, and analysis of 2D and 3D images and supports data import of a large number of standard format files. The grayscale image can be processed simply and effectively by a series of editors and data processing modules provided by the software. Finally, the slices generated by CT can be reconstructed into an accurate digital model and analyzed quantitatively by an efficient algorithm [28]. This study utilizes AVIZO software to assist in tooth segmentation, defining it as a platform for visualization, image preprocessing, manual annotation, 3D reconstruction, and quantitative analysis—not as a fully automated segmentation system. All software functions support researchers’ subjective judgment and precise manual operations. This approach ensures segmentation results leverage the software’s computational and visualization strengths while remaining fully dependent on researchers’ anatomical expertise and subjective assessment. This balance between efficiency and accuracy provides reliable foundational data for subsequent analyses.

### 2.4. Image Calibration

First, we vectorized the 3D model of the mouth and defined the *X*, *Y*, and *Z* axes. *X*, *Y*, and *Z* coordinate axes were established for the 3D tooth model in the AVIZO software interface, which provided an operating interface for the segmentation of tooth, root canal, and alveolar bone in the later stage (Figure 3A); XZ was defined as the coronal plane (Figure 3B); XY was defined as an axial plane (Figure 3C); YZ was defined as a sagittal plane (Figure 3D).

Figure 3Vectorized oral 3D model. (A) Establishment of three‐dimensional coordinates. (B) Coronal plane. (C) Axial plane. (D) Sagittal plane.

**Figure:**
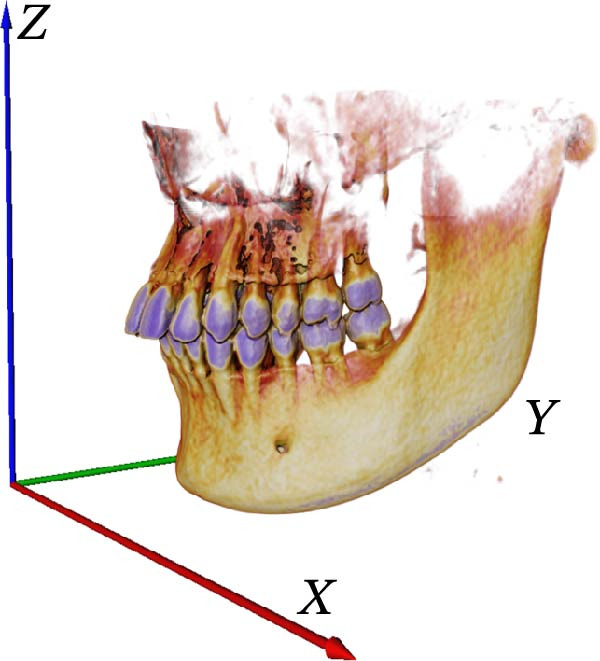
(A)

**Figure:**
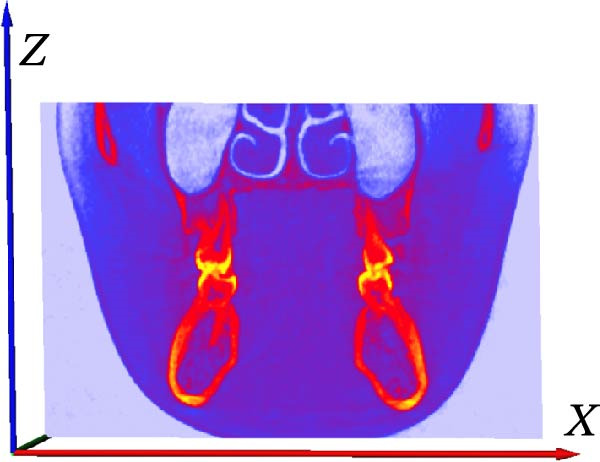
(B)

**Figure:**
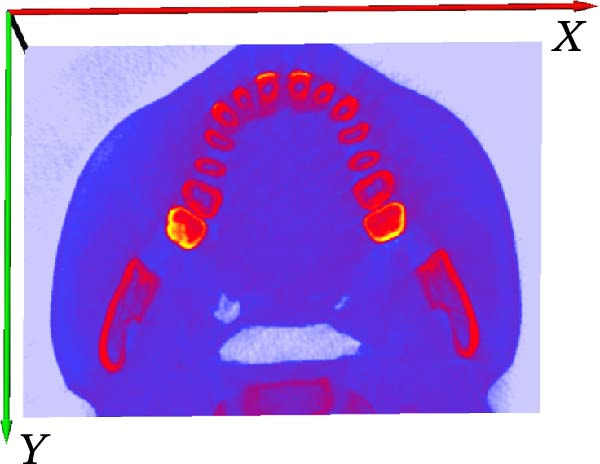
(C)

**Figure:**
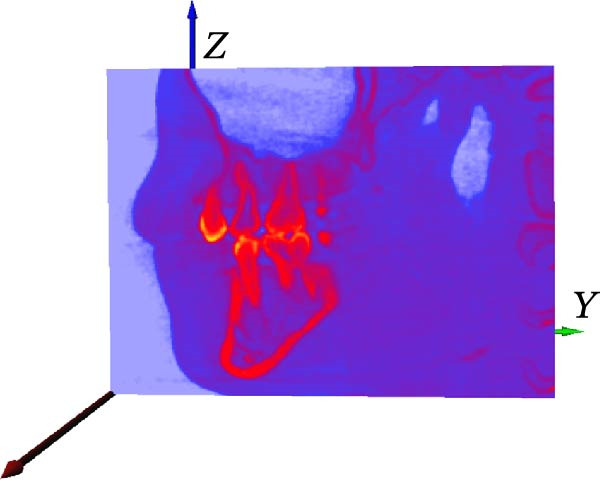
(D)

In order to obtain accurate segmentation and measurement results, the scanned section image needs to be calibrated to determine the accuracy of the model before 3D reconstruction of the image, which was the first step before the tooth segmentation operation. First, all the grayscale slice data were opened through the tool window of AVIZO software, and the grayscale slices were calibrated in length units (Figure 4A). Each grayscale slice was examined for bad dots and irreversible noise, and if bad dots were present, the CBCT needs to be retaken, and then all the slices were stacked and length calibrated (Figure 4B). The volume rendering tool can be used to directly model the block data for the corrected grayscale slice data and establish three‐dimensional coordinate axes in the reconstructed three‐dimensional model (Figure 4C). Axis units were expressed in centimeters. The new block data model was compared with the actual tooth details to ensure that the CBCT data can be segmented and quantified.

Figure 4CBCT image calibration. (A) Original grayscale slice. (B) CBCT grayscale slice overlay. (C) Reconstruction of a 3D tooth model.

**Figure:**
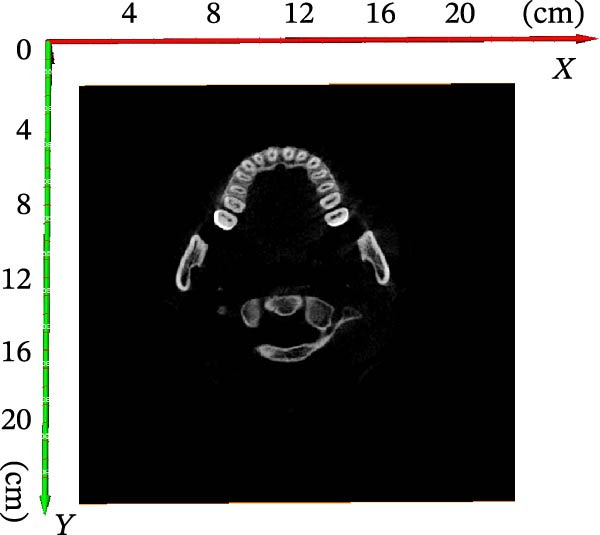
(A)

**Figure:**
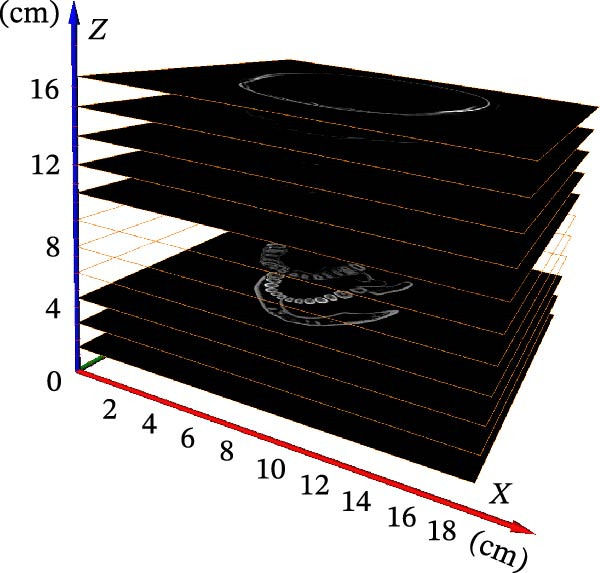
(B)

**Figure:**
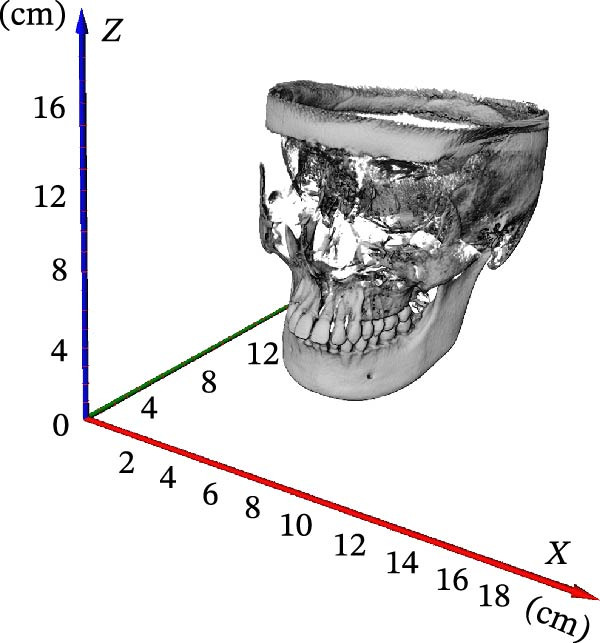
(C)

### 2.5. Image Preprocessing

**Figure 5:**
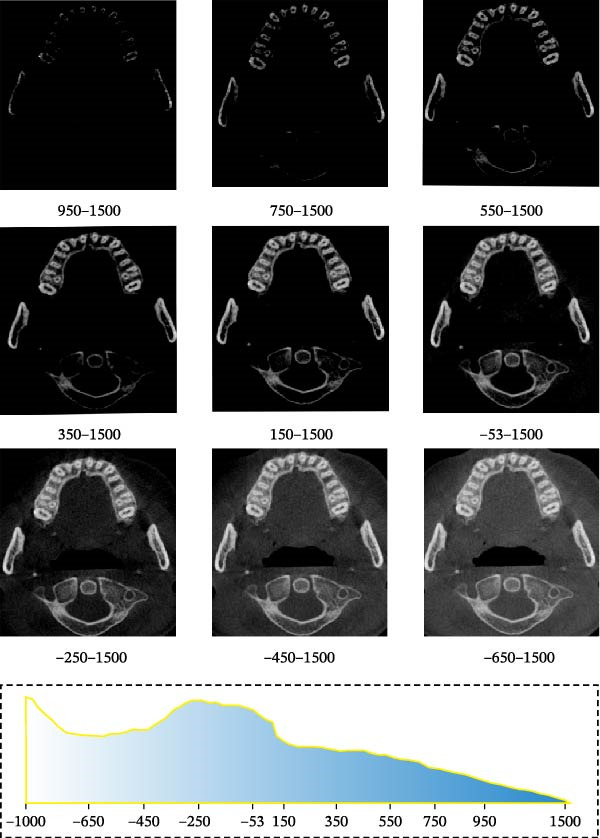
Tooth images in different gray value ranges.

For accurate tooth segmentation, regions with similar gray values need to be separated as much as possible. Therefore, the recognition of an individual tooth was an important preprocessing task in CBCT images. We obtained the best quality grayscale slice images by adjusting the grayscale range and binarization. We adjusted the gray value of the image to the range [−1000, 1500] and preprocessed each tooth image within this gray range. By adjusting the lower threshold of gray value, we could see the image information reflected in different gray ranges (Figure 5). By comparative analysis, it was found that only part of the tooth contour was visible in the grayscale range [950, 1500] and [750, 1500], and the tooth could not be segmented completely in this period. Between the grayscale range [550, 1500, 350, 1500] and [150, 1500], although the complete tooth and root canal structure could be displayed, the complete alveolar bone could not be displayed, and the alveolar bone could not be completely segmented in the later stage. In the grayscale range [−53, 1500, −250, 1500, −450, 1500] and [−650, 1500], the complete tooth, root canal, and alveolar bone structure can be displayed. However, images with a threshold range [−250 to 1500] exhibit greater clarity. Therefore, to ensure precise segmentation of teeth, root canals, and alveolar bone structures in subsequent stages, we selected tooth images within the threshold range [−250, 1500] for further segmentation processing.

### 2.6. Image Noise Reduction and Enhancement

**Figure 6:**
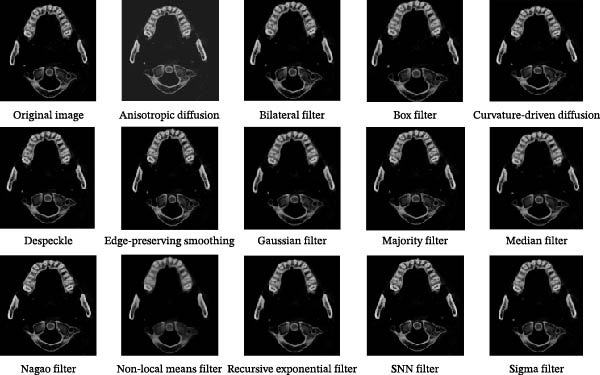
Image noise reduction results of different algorithms.

**Figure 7:**
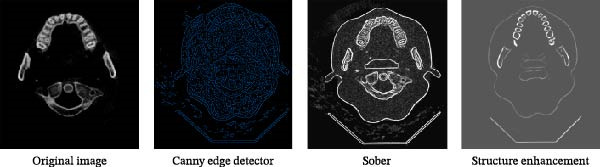
Image enhancement results of different algorithms.

When taking CBCT, it will be affected by the interference of the external environment and the variation of X‐ray intensity, and there will be some noise in the obtained gray slice, which will affect the quality of the slice. The noise of the image can be reduced by filtering to improve the quality of the image. Through edge enhancement, clearer sections can be obtained, and the tooth structure can be better distinguished; thus, it can improve the accuracy of tooth image segmentation. AVIZO software provides a variety of filtering and image edge enhancement methods. We found that the tooth and alveolar bone structure of each tooth image after median filtering processing was the clearest among the many filtering methods. The root canal structure after SNN filtering processing was clearer than all other filtering methods. Therefore, we finally chose median filtering for noise reduction before tooth and alveolar bone segmentation and SNN filtering for noise reduction before root canal segmentation (Figure 6). Median filtering is a nonlinear smoothing technique, which can effectively remove pulse noise. We specifically employed a 3 × 3 square median filter, performing multiple iterations on each voxel (iterating port values). This configuration effectively removes noise while preserving image edge details. SNN filter is a symmetric neighbor mean filter, which is a filter based on the symmetry weighting of the rectangular neighbor window structure. It can obtain the difference between relative regions and different regions, which can make the boundary preservation more flexible and reduce the operation. The window size for the SNN filter was set to 3 × 3 × 3 voxels. This parameter selection achieved an optimal balance between effectively suppressing random noise in the images and preserving critical dental anatomical structures, thereby providing higher‐quality input images for subsequent segmentation. In the results of tooth image processing by different enhancement methods, the tooth contour is clearer than that without enhancement after structure enhancement filter, but this enhancement method has no good effect on root canal, so the above enhancement method can be used for processing before tooth segmentation, as shown in Figure 7.

## 3. Results

**Figure 8:**
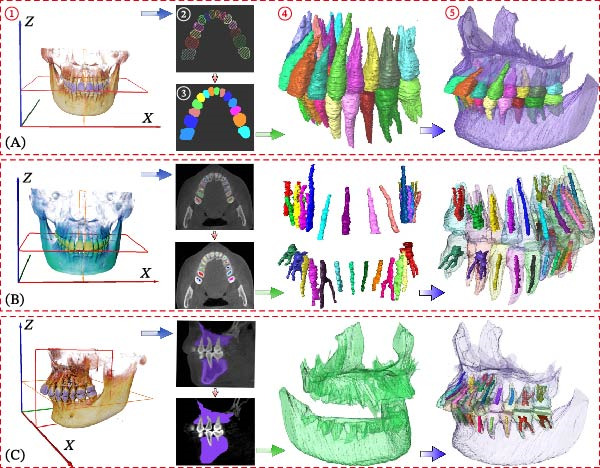
Overall flow chart and 3D visualization of CBCT image. (A) Tooth segmentation and reconstruction. (B) Root canal segmentation and reconstruction. (C) Alveolar bone segmentation and reconstruction.

The CBCT image was segmented after a series of filtering and edge enhancement processing. Image segmentation was a technical process to extract tooth from grayscale sections, which was also the most important link from image processing to 3D reconstruction of tooth grayscale sections. We selected grayscale slices processed by median filtering and edge enhancement by structure enhancement filter for tooth and root canal segmentation. The process and results of manual labeling, segmentation, and 3D reconstruction of tooth are shown in Figure 8A. Tooth was labeled in the XY plane using full manual outlining via the segmentation tool (Figure 1). This relies on tooth outline information to segment individual tooth, and each tooth was represented by a contour line of a different color to identify each tooth (Figure 2). Next, the final segmentation results were superimposed on the original grayscale slices through the slice operation step, which can be overlapped with the original tooth images to compare the segmentation accuracy (Figure 3). Through the volume rendering tool to reconstruct each segmented tooth in three dimensions, we can also clearly distinguish the interface between the upper and lower tooth in the biting (Figure 4). Finally, the reconstructed tooth was visualized in 3D by surface view tool, and the position of different tooth in the oral can be intuitively distinguished by adjusting the transparency (Figure 5). We used the same method described above to manually label and segment the root canal on the XY plane (Figure 8B). Here, we need to emphasize the use of SNN‐filtered processed grayscale slices for root canal segmentation and 3D reconstruction. The grayscale slice processed by the median filter was used for the segmentation and three‐dimensional reconstruction of alveolar bone on the YZ plane. The specific process and results are shown in Figure 8C.

Through the following process, 3D morphological parameters such as 3D volume, shape factor, and angle of teeth can be systematically obtained. By calling the Material Statistics module in AVIZO software and checking the “Volume” option, the 3D volume is directly output. The calculation formula is shown in (1), where *N* is the number of voxel units included.

**Figure 9:**
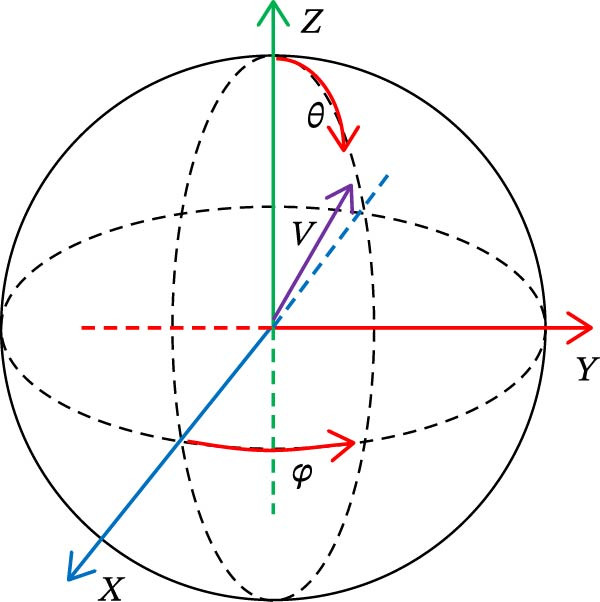
Schematic diagram of dip and azimuth.

The shape factor can indicate the complexity of tooth and root canal morphology; the larger the value, the more complex the tooth and root canal morphology. The form factor calculation formula is shown in (2), where the form factor is represented by SF_3D_, *V*_3D_ is the three‐dimensional volume, and *A*_3D_ is the three‐dimensional surface area, which should be calculated through the surface area module. The dip angle typically refers to the angle of inclination of the tooth’s long axis relative to the axial plane in the sagittal plane (or coronal plane); the azimuth angle refers to its rotational angle relative to the sagittal plane in the axial plane. The diagram of tooth dip and azimuth angle is shown in Figure 9, where θ is the dip and φ is the azimuth.

(1) V3D=N×N0,

(2) SF3D=A3D336πV3D2.

The 3D reconstruction results after tooth segmentation play an important role in digital orthodontics, which can evaluate the location and volume of tooth eruption and root resorption in more detail (Figure 10A) [30]. The results of tooth dip angle and azimuth angle are very important for dentists to make orthodontic plans for patients and can accurately judge the tooth movement angle (Figure 10B) [31]. The results of teeth and alveolar bone segmentation also serve as a valuable tool to provide accurate anatomical information for orthognathic surgery [32, 33]. In addition, in oral implantology, teeth and alveolar bone segmentation facilitates accurate positioning and navigation during surgery, improving the overall success rate of implant restoration [34, 35]. In the clinical diagnosis and treatment of endodontic diseases, an accurate understanding of the complexity of tooth and root canal morphology is an important scientific basis for successful root canal treatment [36]. We can calculate the root canal shape factor. The shape factor can determine the complexity of the root canal system. Generally, the root canal of premolars and molars are more complex, and the value of the form factor of these teeth will be larger. If the tooth has multiple canals and lateral root canals, the form factor is larger (Figure 10C).

Figure 10Calculation results. (A) Three‐dimensional volume size of teeth. (B) Tooth dip and azimuth angle. (C) Tooth and root canal shape factors.

**Figure:**
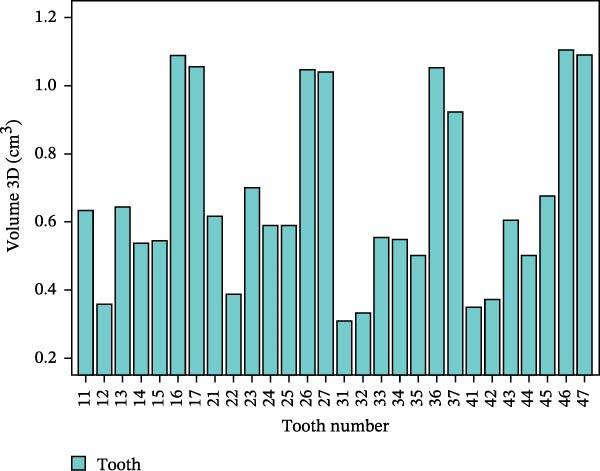
(A)

**Figure:**
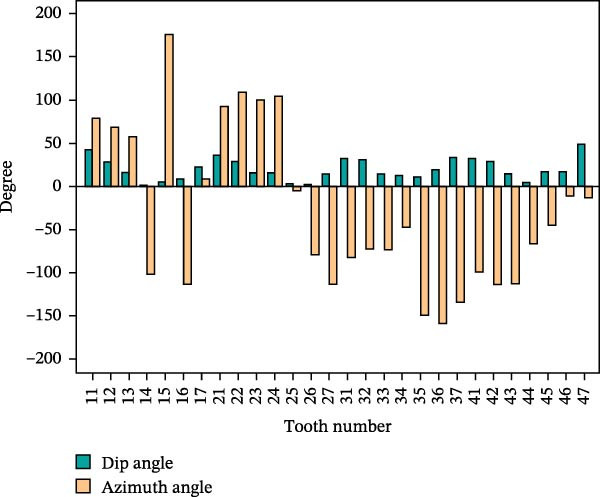
(B)

**Figure:**
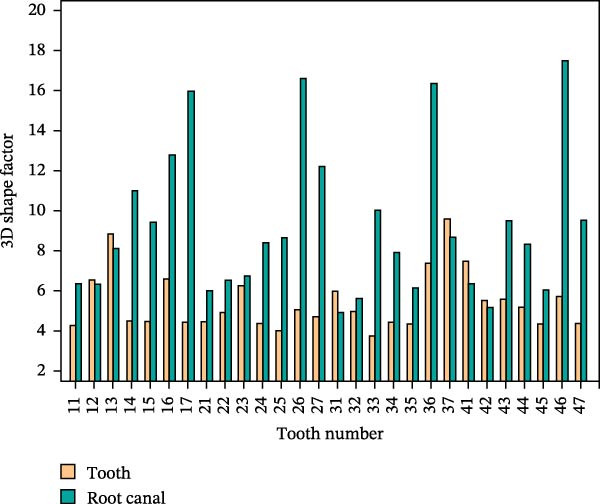
(C)

## 4. Discussion

This study achieves segmentation of teeth, root canals, and alveolar bone structure. The framework of this method is divided into image calibration, noise reduction and enhancement, manual label segmentation, and 3D structure reconstruction. Starting with tooth image calibration, we first determine the three‐dimensional space coordinates and accuracy of the tooth. The results for segmenting individual tooth and root canal depend on the extraction of their contours on the XY plane (axial plane) and alveolar bone contours on the XZ plane (sagittal plane). The complete shape and boundary information of the tooth and root canal can be preserved on the XY plane, and the segmentation accuracy of the tooth and root canal on this plane can be improved. Because there will be blurred boundaries near the root on the YZ and XZ planes, and the overall structure of the tooth and root canal will be incomplete, insufficient and excessive segmentation may occur during the operation. This method of teeth segmentation do not rely on a large number of data to train the network model, which can be applied to the tooth segmentation of different clinical patients.

We can not only realize the root canal segmentation of single tooth, single and multiple root canals, and lateral root canal of single tooth by the above method but also maintain the integrity and continuity of the root canal morphology (Figure 11A). All segmented teeth and root canals have shapes similar to their counterparts (Figure 11B). It is difficult for threshold‐based tooth segmentation algorithms to determine the appropriate threshold size. If the threshold is too small, different structures in the mouth will be divided together (Figure 11C). If the threshold is too large, the reconstructed 3D tooth model will lose part of the information (Figure 11D). Therefore, the tooth model segmented by this method can hardly meet the requirements of modern digital dentistry.

Figure 11Segmentation results. (A) Individual root canal segmentation results. (B) Individual tooth segmentation results. (C) Inadequate segmentation. (D) Excessive segmentation.

**Figure:**
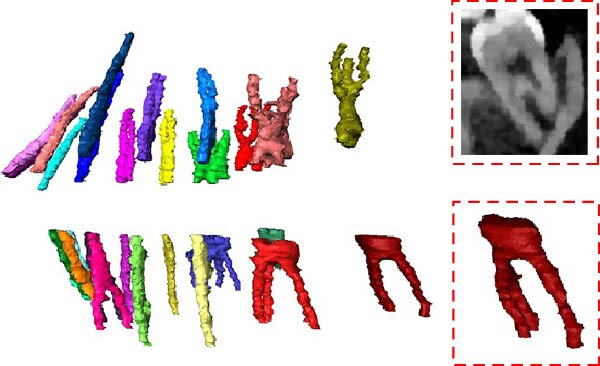
(A)

**Figure:**
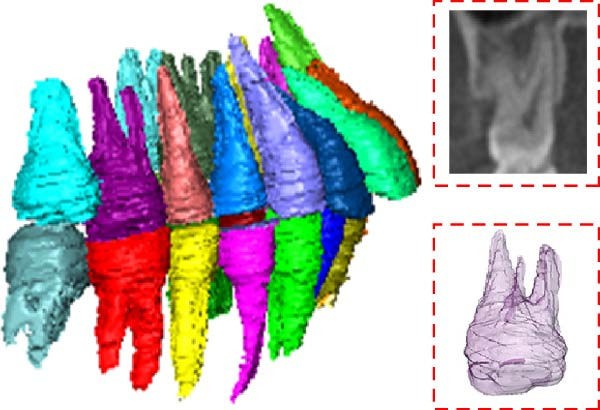
(B)

**Figure:**
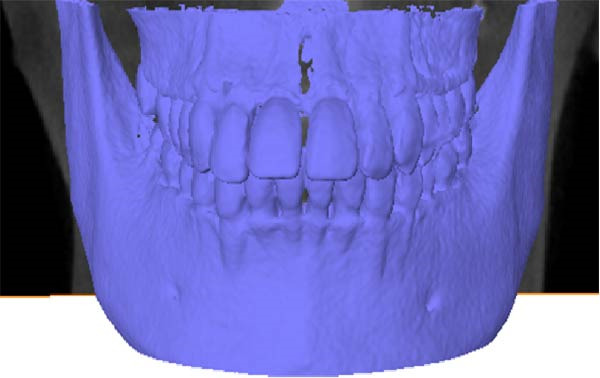
(C)

**Figure:**
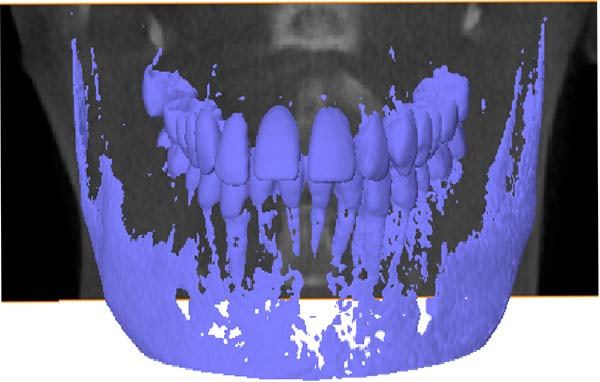
(D)

Based on the AVIZO software platform, this study proposes a method for the manual segmentation of teeth, root canals, and alveolar. The segmentation and 3D reconstruction of each tooth, root canal, and alveolar are achieved through a combination of noise reduction preprocessing applied to CBCT images and meticulous manual annotation. Notably, our segmentation technique does not depend on extensive training datasets, making it adaptable for clinical application across diverse patient populations. This research holds significant scientific merit and clinical applicability, particularly in the design of personalized treatment plans. Image segmentation quality is highly dependent on the operator’s anatomical knowledge and proficiency with the software. For instance, distinguishing cementum from surrounding bone tissue necessitates combining grayscale thresholding with morphological judgment. Novice operators may introduce subjective errors. Time consumption depends on scan resolution, anatomical complexity, and operator experience. Tooth morphology reconstruction typically takes 40 to 60 min; root canal system reconstruction, due to complex anatomy, usually requires 40 to 90 min. In contrast, despite its larger volume, the alveolar bone exhibits more uniform structure, resulting in shorter reconstruction times of 20 to 40 min. While AI‐based automated methods can accelerate the process, manual segmentation allows for more precise adjustments to tissue contrast variations and human interference. AVIZO offers a powerful 3D dental segmentation toolkit with advantages including customizable workflows, exceptional rendering quality, and compatibility with CBCT data. However, it features a learning curve, as manual segmentation requires proficiency with the software’s brush tools. We recommend 10 to 20 h of training to ensure efficient operation.

AVIZO has excelled in research and modeling, but its shortcomings are particularly evident in clinical applications or resource‐limited scenarios. Although the software provides basic algorithms such as threshold segmentation and region growth, the results of segmentation are often unsatisfactory in complex situations (such as crowded dentition, tooth deformities, and root adhesion) and need to rely on manual correction. In addition, segmentation processes are time‐consuming (often hours from data import to completion of corrections), making it difficult to meet clinical immediate diagnostic needs, such as emergency dental surgery planning. However, this workflow can be positioned as a research‐oriented tool and an adjunct to treatment planning. It is important to acknowledge that the imaging workflow optimized in this study was fundamentally designed for and validated on “ideal” dentition, devoid of metallic restorations. Consequently, its performance is expected to be substantially compromised in the presence of severe metal artifacts from sources such as amalgam fillings, metal crowns, or dental implants, where streaking and beam‐hardening effects can obscure critical anatomical boundaries.

Therefore, we plan to combine ITK‐SNAP, 3D Slicer, and other software for preliminary segmentation in future research and then import the results into AVIZO for refining processing. At the same time, external tools (such as TensorFlow and MONAI) will be used to train the U‐Net model of teeth, and the segmentation results will be followed up. In short, AVIZO is better suited as a refined postprocessing tool for tooth segmentation than as an end‐to‐end automation solution. In complex or large sample research, its limitations are particularly significant, so it is necessary to combine other technical means to make up for the shortcomings.

## 5. Conclusions

Based on the AVIZO software platform, this study proposes a method for the manual segmentation of teeth, root canals, and alveolar. The segmentation and 3D reconstruction of each tooth, root canal, and alveolar are achieved through a combination of noise reduction preprocessing applied to CBCT images and meticulous manual annotation. Notably, our segmentation technique does not depend on extensive training datasets, making it adaptable for clinical application across diverse patient populations. This research holds significant scientific merit and clinical applicability, particularly in the design of personalized treatment plans.

## Author Contributions

**Yan Liu:** conceptualization, software. **Chunshen Li:** methodology, validation. **Pengyu Chen:** formal analysis, investigation. **Yunshan Zhao:** resources, data curation. **Liang-qiu-yue Zhong:** writing – original draft preparation. **Zhongyi Xiao:** writing – review and editing. **Xi Chen:** supervision, project administration, funding acquisition.

## Funding

This work was supported by the Oral Flora Changes in Hepatocellular Carcinoma Patients and Periodontitis Accelerates the Malignant Progression of Hepatocellular Carcinoma in Rats (Grant HX202323).

## Disclosure

All authors have read and agreed to the published version of the manuscript.

## Conflicts of Interest

The authors declare no conflicts of interest.

## Data Availability

The data that support the findings of this study are available from the corresponding author upon reasonable request.
